# Human cystic echinococcosis in southwest Iran: a 15-year retrospective epidemiological study of hospitalized cases

**DOI:** 10.1186/s41182-020-00238-3

**Published:** 2020-06-19

**Authors:** Reza Shahriarirad, Amirhossein Erfani, Mehrdad Eskandarisani, Mohammad Rastegarian, Hajar Taghizadeh, Bahador Sarkari

**Affiliations:** 1 Student Research Committee, Shiraz University of Medical Sciences, Shiraz, Iran; 2 Thoracic and Vascular Surgery Research Center, Shiraz University of Medical Sciences, Shiraz, Iran; 3 Department of Parasitology and Mycology, School of Medicine, Shiraz University of Medical Sciences, Shiraz, Iran; 4 Basic Sciences in Infectious Diseases Research Center, Shiraz University of Medical Sciences, Shiraz, Iran

**Keywords:** Cystic echinococcosis, Hospital record, Fars prevalence, Southwestern Iran

## Abstract

**Background:**

Cystic echinococcosis (CE) is considered as a neglected disease with significant mortality and morbidity in most of the developing countries. The current study aimed to retrospectively assess the demographic and epidemiologic features of human CE surgical cases in a 15-year period in Fars province, southwestern Iran.

**Methods:**

A 15-year (2004–2018) retrospective study was conducted to find out the epidemiological and clinical picture of CE in patients who undergone surgeries for CE in two main hospitals in Fars Province, southwestern Iran. Hospital records were reviewed, and data were retrieved from each CE patient’s record.

**Results:**

A total of 501 CE surgical cases were recorded during a 15-year period, corresponding to an average annual incidence of 33.4 and a surgical incidence rate of 0.74/100,000 population. Of these, 242 (48.6%) were male, and 256 (52.4%) were female. Patients’ age ranged from 2 to 96 years, with a mean age of 34.92 (± 19.87) years. A significantly higher rate of CE cases was noted in subject’s ≥ 50 years of age. The highest frequency of cases (62:12.5%) was recorded in the year 2017. The most commonly involved organs were liver (339 cases; 57.8%) and lung (279 cases; 47.6%). Concurrent involvement of two organs was seen in 58 (9.9%) cases of both lung and liver, 10 (1.6%) cases of lung and other locations (but not liver), and 23 (3.9%) cases of liver and other locations (but not lung). Reoperation was noted in 67 (13.4%) of the cases. The size of the lung hydatid cyst varied, ranging between 2 and 24 cm (mean = 7.33, SD = 3.737). The size of liver hydatid cysts ranged from 1 to 26 cm (mean 9.04, SD = 4.275).

**Conclusion:**

The findings of the current study demonstrated a nearly constant prevalence of CE during the last 15 years in southern Iran. Further studies are needed to find out the reasons behind the recurrence of the disease, which is substantial, in surgically-treated patients.

## Background

Cystic echinococcosis (CE), due to *Echinococcus granulosus*, is one of the most significant zoonotic diseases, throughout most parts of the world [1]. Herbivores such as sheep, goats, and swine are intermediate hosts of *E. granulosus*, which become infected by eating the worm eggs passed in the carnivores’ feces. Carnivores, as definitive hosts, ingest the cyst-containing organs of herbivores and harbor worms in their digestive system [2]. Humans become infected via ingesting embryonated eggs through hands, water, or food contaminated with parasite eggs that passed through the feces of definitive hosts. The oncospheres spread through blood and lymphatic circulation to the liver, the lungs, and other organs, where the development of the *Echinococcus* cysts occurs.

Cystic echinococcosis is asymptomatic at the early phases of the infection; however, it becomes symptomatic when the cysts become larger or complicated. Clinical manifestations of CE vary based on the size, location, and condition of the cystic structure [3].

CE is considered a neglected disease with significant mortality and morbidity in most of the developing countries [1, 4, 5 ]. The disease is a major health and economic challenges in the Middle East countries, including Iran where about 1% of all hospital surgeries are accounted for this disease [1, 4, 5]. A recent systematic review and meta-analysis reported the overall seroprevalence of CE in Iran to be 4.2% in human hosts, with the highest seroprevalence (5.8%) in the South and the least (2.2%) in the central areas of the country [6].

CE is more frequently occurring in rural and nomadic communities where people have continuous contact with dogs, the definitive host of *E. granulosus*. Studies in both West and East Azerbaijan and Hamedan provinces in Iran demonstrated that rural residents constitute the most common referral cases of CE [7–9].

CE is one of the most important parasitic diseases in Fars province in the southwest of Iran [5, 10]. Fars province is the center of agriculture and animal husbandry in Iran and one of the most important and populated tribal nomads (Qashqai) resides in this area. The burden and incidence of CE which are the basis for the development of appropriate control programs are poorly known in Fars province, in southwestern Iran. Hence, the current study was designed and conducted to assess the demographic and epidemiological features of CE during a 15-year period in the south of Iran, based on the hospital records.

## Methods

### Study area

This study was conducted in Fars Province in southwestern Iran. The district is located at an altitude of 1545 m above the sea level at geographical coordinates of 29° 36′ 37″ N latitude and 52° 31′ 52″ E longitudes. The area has a hot summer and moderate winter. Agriculture has always been a major part of the economy in Fars province. The province has a population of 4.6 million.

### Data collection

In this retrospective study, hospital records of CE patients were reviewed, and data were retrieved for a 15-year period, from 2004 to 2018 at the main university–affiliated and referral hospitals (Nemazi and Shahid Faghihi) in Shiraz, capital of Fars Province.

Hydatid cyst diagnosis was based on histopathological confirmation after the surgery. Moreover, those patients in which the CE was initially diagnosed by imaging findings and confirmed at surgery, and those with negative histopathological findings (although rare), but characteristic imaging findings and with a positive serology test (mainly CCIEP: Counter Current Immunoelectrophoresis) were considered and recorded as CE cases.

Only cases with a final diagnosis of any type of CE at hospital discharge which were recorded with a unique disease code (based on the ICD-9 and ICD-10: International Classification of Diseases; 122.9, and 122.8 for ICD9; B67.8, K77.0, B67.9, and J99.8 for ICD10) were included in the data analysis. Suspected cases were not included in the study.

From each patients’ hospital record, demographical data (age, sex, place of residence, etc.), history of the previous CE, hydatid cyst features (size, location, multiple organ involvement), treatment measurement (the type of surgery, drug therapy, recurrence), and duration of hospital stay were collected in a data sheet.

The hospital records for a few years of the study were not available in the hospital database. Therefore, the medical records were searched manually by members of the research team which consisted of physicians as well as medical students.

### Statistical analysis

All the statistical analyses were performed by using statistical package for social sciences (SPSS Inc., Chicago, Illinois, USA) version 22.0. A descriptive analysis was performed on the demographics characteristics. Categorical and continuous data were reported as proportions and means ± SDs. Chi-square or Fisher’s exact test was used to test the differences between the categorical variables.

## Results

**Fig. 1:**
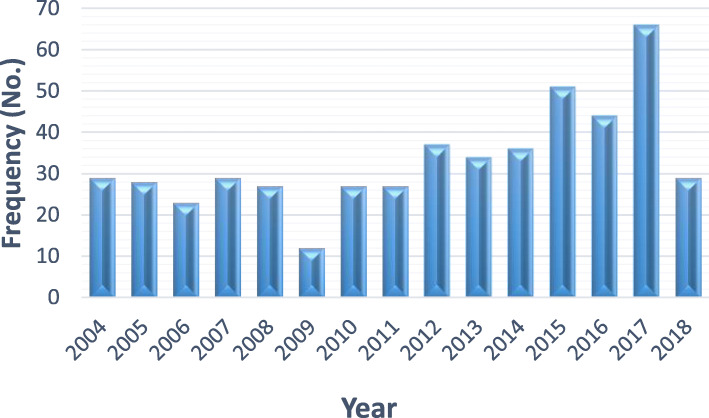
Frequency of CE cases per year, during 2004–2018 in Fars province, southwest Iran

A total of 501 CE surgical cases were diagnosed and underwent surgery in two main hospitals in the 15-year period from 2004 to 2018, giving an average annual incidence of 33.4 cases and an annual incidence rate of 0.74 per 100,000 population. The mean age of the patients was 34.92 (± 19.87) years. The youngest patient was a 2-year-old girl, and the oldest one was a 96-year-old man. Of the total number of patients, 26% were ≥ 50 years old. Out of 501 CE cases, 242 (48.6%) were male while 256 (51.4%) were female. Furthermore, based on the gender of the patients, the median age of male cases was 30 years [IQR 14–48], (mean = 32.79, SD = 20.37), while for female, the median was 35 years [IQR 22–53] (mean = 36.77, SD = 19.31). The highest frequency of cases (62, 12.5%) was recorded in the year 2017. Figure 1 shows the frequency of CE cases, per year in the current study.

Considering the residential areas of the patients, the majority of cases (76%) originating from Fars Province, whereas cases from neighboring Provinces mainly Kohgiluyeh and Boyer-Ahmad (7%) and Bushehr (4.4%), were also among the patients.

The median duration of hospital stay for patients was 7.5 days [IQR 5–13]. The most commonly involved organs were liver (339 cases; 57.8%), and lung (279 cases; 47.6%). Also, 46 (7.8%) cases had hydatid cyst in other locations including spleen, heart, and diaphragm. Among the lung hydatid cyst cases, 126 (64.9%) were located in the right lung while 103 (59.5%) were located in the left lung. Also, regarding the liver hydatid cysts, 193 (47.1%) of cases had cyst in the right lobe while 76 (18.9%) had cyst in the left lobe. Concurrent involvement of two organs was seen in 58 (9.9%) cases of both lung and liver, 10 (1.7%) cases of lung and other locations (excluding liver), and 22 (3.8%) cases of liver and other locations (excluding lung). Table 1 shows the location of hydatid cyst in CE cases in southwest Iran.

**Table 1:** location of hydatid cysts in CE cases in southwestern Iran, based on hospital record, during a 15-year period (2004–2018)

Location	Frequency (No.)	Percent (%)
Lung	279	47.6
Liver	339	57.8
Spleen	14	2.4
Pelvic cavity	7	1.2
Heart	4	0.7
Sub diaphragm	5	0.9
Abdominal and peritoneal cavity	5	0.9
Other locations^*, **^	12	2
Lung and liver	58	9.9
Lung and spleen	2	0.3
Lung and pelvic cavity	1	0.2
Lung and sub diaphragm	2	0.3
Lung and heart	2	0.3
Lung and other locations^*^	3	0.5
Liver and spleen	6	1
Liver and pelvic cavity	6	1
Liver and sub diaphragm	3	0.5
Liver and abdominal and peritoneal cavity	3	0.5
Liver and other locations^**^	5	0.9
Spleen and pelvic cavity	1	0.2

*Common bile duct, mediastina, pancreas, **Kidney, mediastinum, mesothelium of the terminal ileum, pancreas, psoas muscle

Multiple organ involvements of lung, liver, and spleen were seen in 2 (0.3%) cases, lung, liver and pelvic cavity in 1 (0.2%) case, lung liver, and sub-diaphragm in 1 (0.2%) case, and lung, liver, and pancreas in 1 (0.2%) case.

Secondary hydatid cyst or relapse was seen in 67 (13.37%) of the patients, in which the secondary cysts in 28 (41.8%) were lung cysts, 42 (62.7%) were liver cyst, 1 (1.5%) was pelvic cyst, 1 (1.5%) was spleen cyst, 1 (1.5%) was abdominal and peritoneal cavity, and 1 (1.5%) was in the mesothelium of the terminal ileum. It is worth mentioning that 11 of the cases underwent surgery due to tertiary hydatid cyst or relapse for the third time and also 3 of the cases underwent a fourth surgery due to hydatid cyst. Table 2 shows the demographic features of the CE patients along with the cyst features.

**Table 2:** Demographic features of the CE patients along with the cyst characteristics

Characteristics	Frequency (No.)	Percent (%)
Gender		
Male	245	49.1
Female	254	50.9
Province		
Fars	380	75.7
Kohgiluyeh and Boyer-Ahmad	37	7.4
Bushehr	23	4.6
Khuzestan	14	2.8
Foreign countries	6	1.2
Sistan and Balochistan	4	0.8
Kerman	3	0.6
Kermanshah	2	0.4
Khorasan Razavi	2	0.4
Other Provinces^1^	7	1.4
Age groups (years)		
< 10	51	10.2
10–19	70	14.1
20–29	98	19.7
30–39	94	18.9
40–49	55	11
> 50	130	26.1
Number of cysts		
Single	343	69.6
Two cysts	98	19.9
Three cysts	23	4.7
Four cysts	8	1.6
Five or more cysts	21	4.3
Size of cysts (cm)		
= < 5 cm	69	14.1
6–10	164	33.6
11–15	232	47.5
> = 16	23	4.7
Area of the cyst (cm^2^)		
= < 25	179	38.7
26–50	112	24.2
51–75	48	10.4
76–100	62	13.4
> = 100	61	13.2

^1^Other Provinces: Zanjan, Qom, Lorestan, Isfahan, Ilam, Hormozgan, East Azerbaijan

From the 279 cases of lung hydatid cyst, the majority (174: 74%) had a single cyst while 46 (19.6%) cases had two, and 15 (6.4%) cases had three or more cysts. It is worth knowing that from the 279 cases of lung hydatid cyst, 157 (56.3%) cases were male while 120 (43.3%) were female. There was a significant correlation between lung hydatid cysts and the patients’ gender (*P* < 0.001). The size of the lung hydatid cyst varied, ranging between 2 and 24 cm (mean = 7.33, SD = 3.737) with the highest frequency (175, 96.2%) of the largest diameter of the cyst being in group 11 to 15 cm. The lung cyst area ranging from 2 to 490 cm^2^ (mean 42.27, SD = 50.061) with the majority in under 26 cm^2^ group (83:48.3%).

In our study, among the 339 cases of liver hydatid cyst, 194 (68.1%) had single cysts, 56 (19.6%) had two cysts, and 35 (12.3%) had three or more cysts. The size of liver hydatid cysts ranged from 1 to 26 cm (mean 9.04, SD = 4.275) with the highest frequency of the largest diameter of the cyst being in group 6 to 10 cm. The area square centimeters of the liver cyst ranged from 1 to 392 cm^2^ (mean = 58.43, SD = 52.556) with the majority (82: 31.9%) in under 26 cm^2^ group.

In other locations, apart from the lung and liver, the numbers of cysts varied were 21 (3.6%) cases had only a single cyst, 7 (1.2%) cases had two cysts, and 8 (1.4%) cases had three or more. The size of the cysts varied, ranging between 2 and 18 cm (mean = 8.73, SD = 4.527) with the highest frequency (19: 55.9%) being in the 6 to 10 cm group. The area square centimeters of the cysts in other locations ranged between 3 and 314cm^2^ (mean = 59.14, SD = 64.287). Based on the surgical intervention for the patients, 142 (28.3%) underwent radical surgery while 360 (71.9%) underwent conservative surgery. Regarding the medications administered for the patients, 284 (56.6%) patients received anti-helminthic drugs, including albendazole in 282 (56.2%) and mebendazole in 5 (1%) cases.

## Discussion

CE is a zoonotic parasitic infection that occurs all over the world and causes substantial public health problems and economic losses. The Middle East is considered as the hot spot of CE in the world [1, 10]. CE is endemic and highly prevalent in Iran where human cases have been reported from nearly every province in the country [5, 10–12]. The incidence rate of CE in Iran has been reported to be 1.3–3/100,000 population. Our study demonstrated a surgical incidence of 0.74/100,000 inhabitants for CE in southern Iran. It should be noted that the 1.3–3/100,000 incidence of CE has been considered for all CE cases in Iran while our study only included cases that underwent surgery. This means that the total numbers of admitted cases (both surgical and non-surgical cases) in our center could be higher.

A higher rate of CE incidence (4.5 cases/100,000/year) has been reported in neighboring Iraq [13]. An incidence of 0.87 to 6.6 per 100,000 inhabitants has been reported for CE in Turkey [14]. A retrospective analysis of CE in Aydın Adnan Menderes University training and research hospital in Turkey, from 2005 to 2017, reported 247 pathologically confirmed cases [15]. In Pakistan, data regarding human CE is limited [16]. A retrospective study of hospital records from five major metropolitan cities of Pakistan reported 188 surgically confirmed cases of CE corresponding to an annual frequency of 18.8 cases/year [17]. Another retrospective study in Northeastern Punjab Province reported a total of 198 cases of CE during a 6-year period (2012–2017) [18].

Although cases of CE are being reported from all the 31 provinces of Iran, the disease is more prevalent in sheep-breeding areas, including Fars province in the south of the country. The present study describes a detailed status of the geographical distribution as well as the epidemiological features of CE in southern Iran through retrospective reviewing of the patients’ hospital records during a 15-year period. The annual surgical cases of CE were found to be 33.5 cases. It should be noted that some of the CE patients may seek treatment in local hospitals, where sufficient equipment are available for CE operation. It is also worth mentioning that there is a tendency by most surgeons to manage the CE by using a kind of “watch and wait” policy or chemotherapy instead of surgery and only the complicated cases undergoing operations. This again results in a decreased number of patients who undergo surgeries and decrease the number of CE hospital records. With these in mind, the actual annual prevalence rate of CE would be higher in the study area.

CE is reported to have a higher prevalence rate in women than in men in Iran which can be due to the household activities of women that are traditionally a part of their daily routine life [7, 19]. This finding is in line with our previous study and also a retrospective hospital-based study by Abdulhameed in Basrah, Iraq, a neighboring country to Iran, where females were reported to be the main victims of CE [13, 20]. Previous studies suggested that women have the highest chance of contact with sources of infection such as dogs, soil, and vegetable [5, 21, 22]. Housewives, particularly in rural areas, where the disease is more prevalent, have the highest chance of contact with the sources of infection. However, in studies of Cohen et al. [23] and Qaqish et al. [24], such associations were not observed.

In this regard, a meta-analysis by Khalkhali et al. on the prevalence of hydatid cysts in Iran found that although the rate of infection with hydatid cysts was higher in females than males, the difference was not statistically significant [6]. Thus, in some areas, a greater frequency of CE in males may be seen [25–27].

In our study, the majority of CE cases were ≥ 50 years old. This is mainly because CE is a slow-growing cyst in humans and usually presents the signs and symptoms at a higher age of life, whereas the infection may occur in younger people or even teenagers. The fact that all age groups, including children and young adults, were well represented in the confirmed CE cases and suggest that both adults and children are susceptible to infection, as previously documented [5, 28]. In accordance with the available evidence, CE in Iran has no privilege for age. In a study by Moldovan et al., a total of 190 CE cases in two counties of Romania were reviewed and revealed the highest rate of infection in 60–69 years old patients [29].

CE is not uncommon in young children as 51 (10.16%) of cases in our study were children under 10 years old. In line with our findings, a retrospective study regarding CE cases in children in one of the hospitals in the south of Iran reported 57 children with CE during a 12-year period, 2003–2014 [28].

Findings of the present study further documented that hydatid cyst occurs predominantly in the liver and lung and occasionally in the heart, spleen, abdomen, and pelvis. Moreover, the lungs are predominantly infected with hydatid cyst other than any organ possibly due to the presence of greater capillary beds in the lungs in comparison with other organs [30–32].

In the present study, data about the WHO ultrasound classification of the hydatid cysts were not available in the patients’ hospital records. Such data are very important for management as well as follow-up of patients. With the establishment of a regular registration system, such information would be included by radiologists to the patients’ hospital records.

The retrospective hospital surveys have been generally criticized for not providing precise estimates of the disease incidence as not all hospitals in a particular region or district are included in the study. Besides, retrospective hospital survey data on human CE cannot give an accurate picture of the prevalence of the infection. A certain number of cases are not observed in hospital records since the infection is asymptomatic or does not need surgical intervention. Besides, some data are not available in the hospital records. Yet, the hospital records can indicate the public health importance of the disease and, when done constantly over many years, can detect regional variations in the incidence of the infection [33]. More importantly, establishing a registered database for documenting cases of CE in endemic areas such as Iran is essential to reach a better understanding of the prevalence and extent of the disease. Such a system has recently been launched in a few provinces in Iran. Moreover, Iran and Turkey have recently joined the European Register of CE.

## Conclusion

Taken together, findings of the current study revealed that human CE is a common infectious disease in the southwest of Iran with a relatively constant rate during the last 15-year period of evaluation. A health education program to increase awareness of how CE is transmitted, along with regular surveillance of the disease, would help to reduce the infection rate in this area of Iran. Moreover, the burden of disease in the investigated area would justify starting a well-organized control program as CE is a preventable disease.

## Data Availability

SPSS data of the participant can be requested from the authors. Please write to the corresponding author if you are interested in such data.
